# (±)-*trans*-6,6′-Dieth­oxy-2,2′-[cyclo­hexane-1,2-diylbis(nitrilo­methanylyl­idene)]diphenol monohydrate

**DOI:** 10.1107/S1600536814000713

**Published:** 2014-01-22

**Authors:** Nithya Mohan, S. S. Sreejith, M. Sithambaresan, M. R. Prathapachandra Kurup

**Affiliations:** a Department of Applied Chemistry, Cochin University of Science and Technology, Kochi 682 022, India; b Department of Chemistry, Faculty of Science, Eastern University, Sri Lanka, Chenkalady, Sri Lanka

## Abstract

In the title hydrate, C_24_H_30_N_2_O_4_·H_2_O, the organic mol­ecule adopts an *E* conformation with respect to the azomethine double bonds. The cyclo­hexane ring is in a chair conformation. The dihedral angle between benzene rings is 79.6 (2)°. Two intra­molecular O—H⋯N hydrogen bonds are present. In the crystal, the components are linked by O–H⋯O hydrogen bonds and weak C—H⋯π inter­actions, generating a three-dimensional supramolecular architecture.

## Related literature   

For applications of Schiff bases, see: Franceschi *et al.* (1999 ▶); Hwang *et al.* (1998 ▶); Popović *et al.* (2002 ▶); Jones *et al.* (1979 ▶). For a related structure, see: Ambili *et al.* (2012 ▶). For the synthesis of Schiff bases, see: Tümer (2000 ▶). For ring puckering analysis, see: Cremer & Pople (1975 ▶).
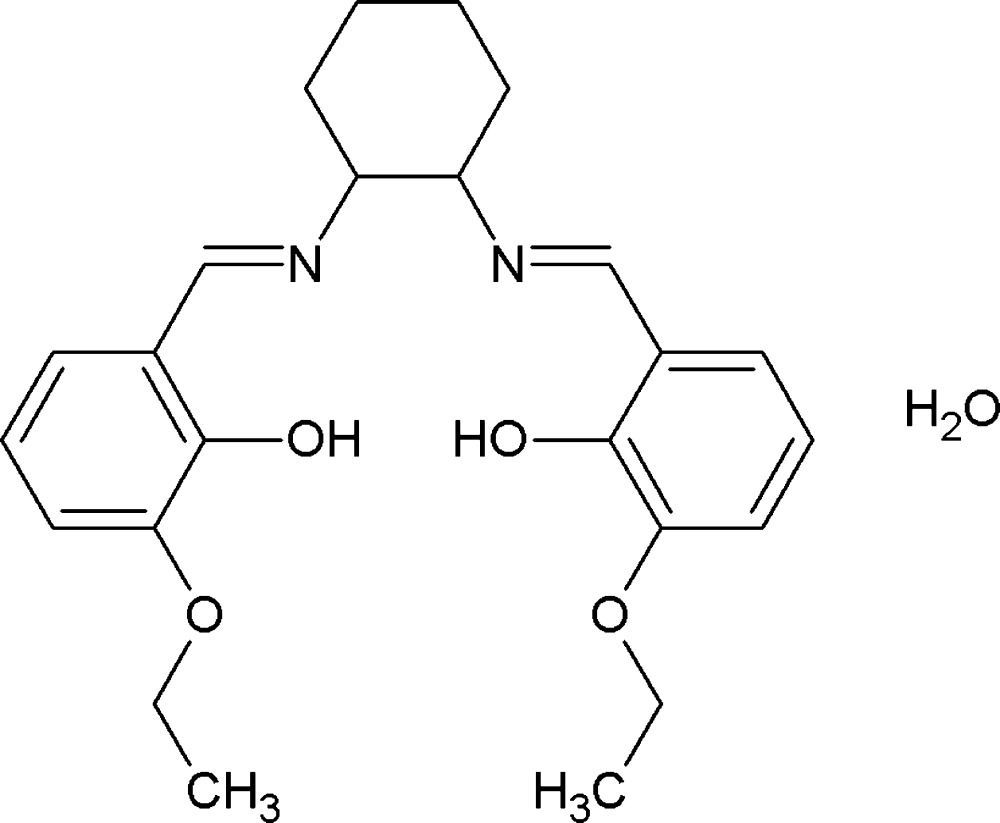


## Experimental   

### 

#### Crystal data  

C_24_H_30_N_2_O_4_·H_2_O
*M* *_r_* = 428.52
Monoclinic, 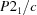

*a* = 9.8241 (18) Å
*b* = 11.6975 (19) Å
*c* = 21.881 (4) Å
β = 111.144 (8)°
*V* = 2345.2 (7) Å^3^
*Z* = 4
Mo *K*α radiation
μ = 0.09 mm^−1^
*T* = 293 K
0.40 × 0.20 × 0.20 mm

#### Data collection  

Bruker APEXII CCD diffractometer
Absorption correction: multi-scan (*SADABS*; Bruker, 2004 ▶) *T*_min_ = 0.977, *T*_max_ = 0.980
16141 measured reflections
5586 independent reflections
2701 reflections with *I* > 2σ(*I*)
*R*_int_ = 0.048

#### Refinement  

*R*[*F* ^2^ > 2σ(*F* ^2^)] = 0.068
*wR*(*F* ^2^) = 0.190
*S* = 1.01
5586 reflections
299 parameters
5 restraints
H atoms treated by a mixture of independent and constrained refinement
Δρ_max_ = 0.24 e Å^−3^
Δρ_min_ = −0.21 e Å^−3^

### 

Data collection: *APEX2* (Bruker, 2004 ▶); cell refinement: *APEX2* and *SAINT* (Bruker, 2004 ▶); data reduction: *SAINT* and *XPREP* (Bruker, 2004 ▶); program(s) used to solve structure: *SHELXS97* (Sheldrick, 2008 ▶); program(s) used to refine structure: *SHELXL97* (Sheldrick, 2008 ▶); molecular graphics: *ORTEP-3 for Windows* (Farrugia, 2012 ▶) and *DIAMOND* (Brandenburg, 2010 ▶); software used to prepare material for publication: *SHELXL97* and *publCIF* (Westrip, 2010 ▶).

## Supplementary Material

Crystal structure: contains datablock(s) I, global. DOI: 10.1107/S1600536814000713/fj2654sup1.cif

Structure factors: contains datablock(s) I. DOI: 10.1107/S1600536814000713/fj2654Isup2.hkl

CCDC reference:

Additional supporting information:  crystallographic information; 3D view; checkCIF report

## Figures and Tables

**Table 1:** Hydrogen-bond geometry (Å, °) *Cg* is the centroid of the C15–C20 ring.

*D*—H⋯*A*	*D*—H	H⋯*A*	*D*⋯*A*	*D*—H⋯*A*
O3—H3′⋯N2	0.85 (1)	1.77 (3)	2.550 (4)	152 (3)
O2—H2′⋯N1	0.84 (1)	1.85 (2)	2.584 (3)	144 (4)
O1*W*—H1*B*⋯O3	0.86 (5)	2.34 (7)	3.005 (5)	134 (8)
O1*W*—H1*B*⋯O4	0.86 (5)	2.38 (8)	3.052 (5)	135 (6)
C21—H21*B*⋯*Cg*	0.97	2.92	3.810 (4)	153

**Fig. 1.**
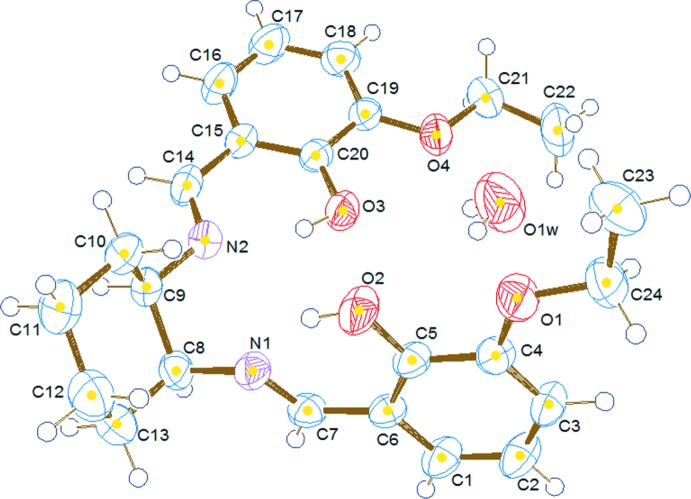
ORTEP diagram of the unique part of the compound, drawn with 50% probability displacement ellipsoids for the non-H atoms.

**Fig. 2.**
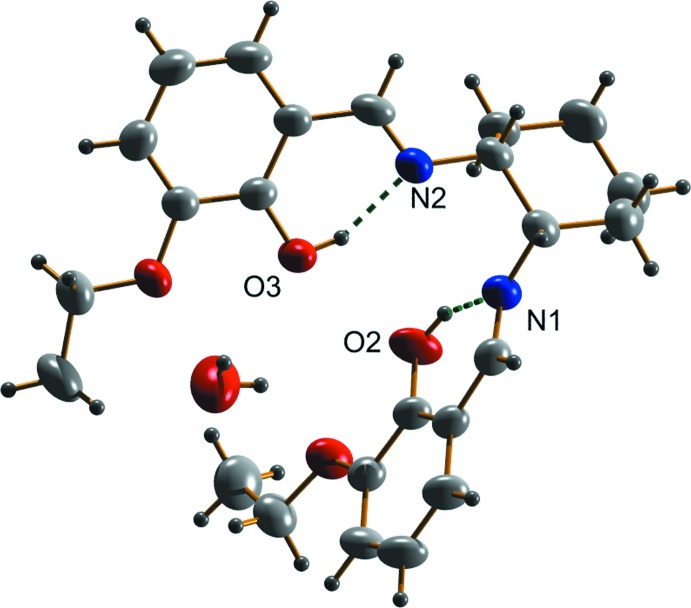
Intramolecular hydrogen bonds present in the compound.

**Fig. 3.**
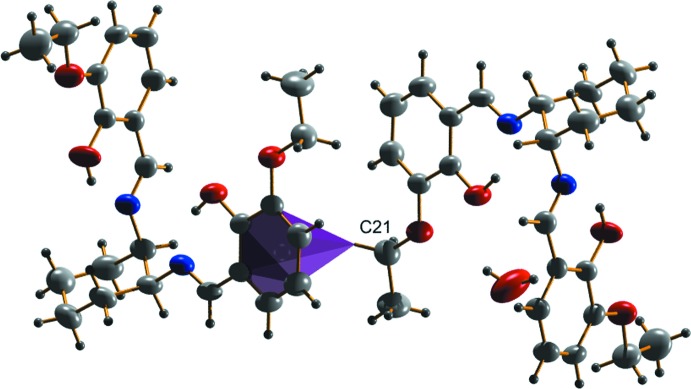
C—H···π interactions found in the title compound.

**Fig. 4.**
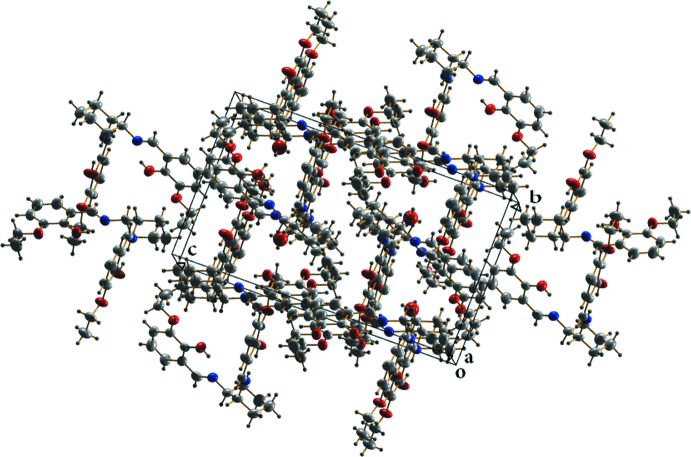
Packing diagram of the compound viewed along a axis.

## supplementary crystallographic information

### 1. Comment

Schiff bases are an important class of ligands in molecular design devoted to energy storage such as molecular batteries (Franceschi, *et al.* 1999) and also in transition metal catalysis (Hwang, *et al.* 1998). Schiff base having intramolecular hydrogen bonding shows photophysical properties such as thermochromism and photochromism (Popović, *et al.* 2002). Schiff bases also have an ability to reversibly bind oxygen (Jones, *et al*.1979).

The title compound crystallizes in the monoclinic, *P*2_1_/c space group. The bond lengths and the bond angles agree with the related structure (Ambili, *et al.*, 2012). The torsional angle, 177.9 (3)° of the azomethine linkage, C9—N2—C14—C15 reveals that the title compound adopts *E* conformation (Fig. 1). The mean plane deviation calculations show that the molecule as a whole is non-planar. Ring puckering analysis (Cremer & Pople, 1975) and least square plane calculations show that the cyclohexyl ring adopts a chair conformation (Q_T_ = 0.565 (4) Å) with the equatorial substitution at C9 for N2 and axial substitution at C8 for N1.

Crystal system consists of intramolecular hydrogen bonds of lengths 1.79 (3) Å and 1.85 (3) Å which exists between the azomethine N atom and the neighbouring phenolic O atom leading to the formation of two six membered rings comprising of atoms C14—C15—C20—O3—H3'···N2 and C7—C6—C5—O2—H2'···N1 (Fig. 2). A C—H···π interaction between one of the hydrogen attached at C21 and the aromatic ring (C15—C20) with H···*Cg* distance of 2.92 Å (Fig. 3) dominates the packing of molecules in the lattice. Fig. 4 shows the packing diagram of the title compound along *a* axis.

### 2. Experimental

The title compound was prepared by following the reported procedure (Tümer, 2000). 3-Ethoxy-2-hydroxybenzaldehyde (0.166 g,1 mmol) was dissolved in ethanol and an ethanolic solution of 1, 2-diaminocyclohexane (0.044 g, 0.5 mmol) was added to it. The mixture was refluxed for 5 h. Slow evaporation of the solution yielded 0.183 g (95%) yellow block type crystals of (±)-*trans*-6,6'-Diethoxy-2,2'-[cyclohexane-1,2-diylbis(nitrilomethanylylidene)]diphenol monohydrate. The compound melts at 115 °C.

IR (KBr, \v in cm^-1^): 1626, 3530, 2930, 1468, 1249 ^1^H NMR (400 MHz, CDCl_3_, δ in p.p.m.): 13.871 (s, 2H), 8.231 (s, 2H), 4.093–4.041 (q, 4H), 1.442–1.477 (t, 6H), 6.679–6.686 (m, 6*H*), 1.589–1.953 (m, 10*H*)

### 3. Refinement

All H atoms on C were placed in calculated positions, guided by difference maps, with C—H bond distances 0.93–0.97 Å. H atoms were assigned as *U*_iso_=1.2Ueq (1.5 for Me). The O bound H atoms were located in a difference Fourier map and their *U*_iso_ values tied to 1.5 times of O5 atom. The O–H distances of water molecule are restrained by *DFIX* and DANG instructions. The phenolic O–H distances, O2–H2' and O3–H3' were restrained to 0.084±0.001 Å. Omitting owing to bad disagreement were the reflections (0 0 2), (1 1 0) and (1 1 1).

### Crystal data

**Table:**

C_24_H_30_N_2_O_4_·H_2_O	*F*(000) = 920
*M**_r_* = 428.52	*D*_x_ = 1.214 Mg m^−^^3^
Monoclinic, *P*2_1_/*c*	Mo *K*α radiation, λ = 0.71073 Å
Hall symbol: -P 2ybc	Cell parameters from 3026 reflections
*a* = 9.8241 (18) Å	θ = 2.8–23.5°
*b* = 11.6975 (19) Å	µ = 0.09 mm^−^^1^
*c* = 21.881 (4) Å	*T* = 293 K
β = 111.144 (8)°	Block, yellow
*V* = 2345.2 (7) Å^3^	0.40 × 0.20 × 0.20 mm
*Z* = 4	

### Data collection

**Table:**

Bruker APEXII CCD diffractometer	5586 independent reflections
Radiation source: fine-focus sealed tube	2701 reflections with *I* > 2σ(*I*)
Graphite monochromator	*R*_int_ = 0.048
Detector resolution: 8.33 pixels mm^-1^	θ_max_ = 28.0°, θ_min_ = 2.4°
ω and φ scan	*h* = −12→12
Absorption correction: multi-scan (*SADABS*; Bruker, 2004)	*k* = −15→15
*T*_min_ = 0.977, *T*_max_ = 0.980	*l* = −28→28
16141 measured reflections	

### Refinement

**Table:**

Refinement on *F*^2^	Secondary atom site location: difference Fourier map
Least-squares matrix: full	Hydrogen site location: inferred from neighbouring sites
*R*[*F*^2^ > 2σ(*F*^2^)] = 0.068	H atoms treated by a mixture of independent and constrained refinement
*wR*(*F*^2^) = 0.190	*w* = 1/[σ^2^(*F*_o_^2^) + (0.0561*P*)^2^ + 1.8063*P*] where *P* = (*F*_o_^2^ + 2*F*_c_^2^)/3
*S* = 1.01	(Δ/σ)_max_ < 0.001
5586 reflections	Δρ_max_ = 0.24 e Å^−^^3^
299 parameters	Δρ_min_ = −0.21 e Å^−^^3^
5 restraints	Extinction correction: *SHELXL97* (Sheldrick, 2008), Fc^*^=kFc[1+0.001xFc^2^λ^3^/sin(2θ)]^-1/4^
Primary atom site location: structure-invariant direct methods	Extinction coefficient: 0.0022 (8)

### Special details

**Table:**

Geometry. All e.s.d.'s (except the e.s.d. in the dihedral angle between two l.s. planes) are estimated using the full covariance matrix. The cell e.s.d.'s are taken into account individually in the estimation of e.s.d.'s in distances, angles and torsion angles; correlations between e.s.d.'s in cell parameters are only used when they are defined by crystal symmetry. An approximate (isotropic) treatment of cell e.s.d.'s is used for estimating e.s.d.'s involving l.s. planes.
Refinement. Refinement of *F*^2^ against ALL reflections. The weighted *R*-factor *wR* and goodness of fit *S* are based on *F*^2^, conventional *R*-factors *R* are based on *F*, with *F* set to zero for negative *F*^2^. The threshold expression of *F*^2^ > σ(*F*^2^) is used only for calculating *R*-factors(gt) *etc*. and is not relevant to the choice of reflections for refinement. *R*-factors based on *F*^2^ are statistically about twice as large as those based on *F*, and *R*- factors based on ALL data will be even larger.

### Fractional atomic coordinates and isotropic or equivalent isotropic displacement parameters (Å^2^)

**Table:**

	*x*	*y*	*z*	*U*_iso_*/*U*_eq_	
O1	0.7073 (2)	0.08906 (17)	0.34718 (12)	0.0591 (6)	
O2	0.8516 (2)	0.28131 (19)	0.35839 (14)	0.0629 (7)	
O3	0.7204 (3)	0.4164 (2)	0.18225 (11)	0.0538 (6)	
O4	0.5589 (2)	0.32967 (18)	0.06864 (10)	0.0547 (6)	
N1	0.8533 (3)	0.5022 (2)	0.35838 (12)	0.0449 (6)	
N2	0.9286 (3)	0.5466 (2)	0.24970 (12)	0.0466 (6)	
C1	0.4960 (3)	0.3961 (3)	0.34503 (16)	0.0508 (8)	
H1	0.4499	0.4658	0.3442	0.061*	
C2	0.4199 (4)	0.2961 (3)	0.34086 (17)	0.0574 (9)	
H2	0.3232	0.2984	0.3380	0.069*	
C3	0.4869 (3)	0.1916 (3)	0.34092 (15)	0.0496 (8)	
H3	0.4339	0.1243	0.3369	0.060*	
C4	0.6311 (3)	0.1869 (2)	0.34687 (14)	0.0420 (7)	
C5	0.7104 (3)	0.2888 (2)	0.35266 (14)	0.0400 (7)	
C6	0.6415 (3)	0.3937 (2)	0.35044 (13)	0.0382 (7)	
C7	0.7186 (3)	0.5000 (2)	0.35060 (14)	0.0424 (7)	
H7A	0.6677	0.5686	0.3448	0.051*	
C8	0.9215 (3)	0.6119 (2)	0.35447 (16)	0.0471 (8)	
H8	0.8450	0.6678	0.3328	0.056*	
C9	1.0171 (3)	0.5952 (3)	0.31316 (15)	0.0462 (8)	
H9	1.0533	0.6701	0.3058	0.055*	
C10	1.1468 (3)	0.5191 (3)	0.34789 (16)	0.0539 (8)	
H10A	1.2077	0.5135	0.3217	0.065*	
H10B	1.1125	0.4430	0.3524	0.065*	
C11	1.2369 (4)	0.5653 (3)	0.41526 (18)	0.0676 (10)	
H11A	1.2790	0.6384	0.4106	0.081*	
H11B	1.3162	0.5129	0.4369	0.081*	
C12	1.1432 (4)	0.5804 (3)	0.45706 (18)	0.0711 (11)	
H12A	1.1098	0.5063	0.4657	0.085*	
H12B	1.2013	0.6145	0.4987	0.085*	
C13	1.0126 (4)	0.6560 (3)	0.42242 (18)	0.0642 (10)	
H13A	1.0466	0.7326	0.4185	0.077*	
H13B	0.9517	0.6606	0.4487	0.077*	
C14	0.9536 (3)	0.5704 (3)	0.19771 (16)	0.0497 (8)	
H14	1.0277	0.6217	0.2002	0.060*	
C15	0.8699 (3)	0.5199 (3)	0.13530 (14)	0.0426 (7)	
C16	0.9044 (4)	0.5437 (3)	0.07977 (17)	0.0597 (9)	
H16	0.9810	0.5929	0.0830	0.072*	
C17	0.8259 (4)	0.4948 (3)	0.02104 (17)	0.0640 (10)	
H17	0.8501	0.5100	−0.0155	0.077*	
C18	0.7098 (4)	0.4224 (3)	0.01536 (15)	0.0535 (8)	
H18	0.6567	0.3899	−0.0250	0.064*	
C19	0.6726 (3)	0.3985 (2)	0.06895 (15)	0.0419 (7)	
C20	0.7557 (3)	0.4444 (2)	0.13042 (14)	0.0403 (7)	
C21	0.4662 (4)	0.2842 (3)	0.00700 (16)	0.0575 (9)	
H21A	0.5221	0.2365	−0.0117	0.069*	
H21B	0.4222	0.3458	−0.0235	0.069*	
C22	0.3504 (4)	0.2149 (3)	0.0195 (2)	0.0823 (12)	
H22A	0.2975	0.2627	0.0389	0.124*	
H22B	0.3951	0.1532	0.0487	0.124*	
H22C	0.2844	0.1846	−0.0212	0.124*	
C23	0.7313 (5)	−0.1081 (3)	0.3322 (2)	0.0803 (12)	
H23A	0.8103	−0.1114	0.3737	0.120*	
H23B	0.7691	−0.0918	0.2983	0.120*	
H23C	0.6814	−0.1803	0.3234	0.120*	
C24	0.6272 (4)	−0.0162 (3)	0.33377 (19)	0.0616 (9)	
H24A	0.5469	−0.0118	0.2920	0.074*	
H24B	0.5878	−0.0319	0.3677	0.074*	
O1W	0.5867 (5)	0.1883 (3)	0.1898 (2)	0.1125 (13)	
H3'	0.786 (3)	0.447 (3)	0.2150 (13)	0.108 (17)*	
H2'	0.890 (4)	0.3467 (17)	0.362 (2)	0.091 (14)*	
H1A	0.635 (10)	0.203 (8)	0.2306 (13)	0.32 (6)*	
H1B	0.573 (11)	0.255 (3)	0.172 (4)	0.31 (5)*	

### Atomic displacement parameters (Å^2^)

**Table:**

	*U*^11^	*U*^22^	*U*^33^	*U*^12^	*U*^13^	*U*^23^
O1	0.0575 (15)	0.0360 (12)	0.0902 (17)	0.0028 (11)	0.0343 (13)	−0.0022 (11)
O2	0.0410 (13)	0.0402 (14)	0.118 (2)	0.0047 (11)	0.0407 (14)	−0.0023 (14)
O3	0.0549 (14)	0.0642 (15)	0.0469 (13)	−0.0202 (12)	0.0240 (12)	−0.0055 (12)
O4	0.0559 (14)	0.0564 (14)	0.0502 (13)	−0.0196 (11)	0.0172 (11)	−0.0078 (11)
N1	0.0388 (15)	0.0399 (14)	0.0600 (16)	0.0007 (12)	0.0225 (13)	−0.0034 (12)
N2	0.0411 (15)	0.0477 (15)	0.0517 (16)	−0.0072 (12)	0.0175 (13)	−0.0053 (12)
C1	0.0396 (18)	0.0480 (18)	0.071 (2)	0.0099 (15)	0.0274 (17)	0.0086 (16)
C2	0.0392 (18)	0.062 (2)	0.078 (2)	0.0021 (17)	0.0295 (17)	0.0112 (18)
C3	0.0453 (19)	0.0455 (18)	0.062 (2)	−0.0063 (15)	0.0246 (16)	0.0087 (15)
C4	0.0448 (18)	0.0382 (16)	0.0466 (17)	0.0036 (14)	0.0207 (14)	0.0044 (13)
C5	0.0339 (16)	0.0414 (16)	0.0485 (17)	0.0059 (13)	0.0193 (14)	0.0030 (14)
C6	0.0351 (16)	0.0401 (16)	0.0422 (16)	0.0070 (13)	0.0173 (13)	0.0034 (13)
C7	0.0432 (18)	0.0379 (16)	0.0493 (18)	0.0074 (14)	0.0204 (15)	−0.0012 (14)
C8	0.0428 (18)	0.0366 (16)	0.063 (2)	−0.0031 (14)	0.0207 (16)	−0.0086 (15)
C9	0.0417 (18)	0.0417 (17)	0.0575 (19)	−0.0112 (14)	0.0206 (15)	−0.0078 (15)
C10	0.0363 (18)	0.060 (2)	0.068 (2)	−0.0026 (16)	0.0219 (16)	−0.0094 (17)
C11	0.045 (2)	0.079 (3)	0.071 (2)	−0.0057 (19)	0.0107 (18)	0.000 (2)
C12	0.069 (3)	0.080 (3)	0.060 (2)	−0.015 (2)	0.017 (2)	−0.009 (2)
C13	0.071 (2)	0.056 (2)	0.074 (2)	−0.0132 (19)	0.036 (2)	−0.0205 (19)
C14	0.0391 (18)	0.0450 (18)	0.068 (2)	−0.0075 (14)	0.0234 (16)	0.0032 (16)
C15	0.0343 (16)	0.0479 (17)	0.0481 (18)	−0.0030 (14)	0.0178 (14)	−0.0008 (14)
C16	0.051 (2)	0.073 (2)	0.062 (2)	−0.0134 (18)	0.0286 (18)	0.0062 (18)
C17	0.061 (2)	0.086 (3)	0.054 (2)	−0.009 (2)	0.0308 (19)	0.005 (2)
C18	0.053 (2)	0.064 (2)	0.0451 (18)	0.0004 (17)	0.0188 (16)	−0.0038 (16)
C19	0.0383 (17)	0.0400 (16)	0.0498 (18)	0.0002 (14)	0.0187 (14)	−0.0014 (14)
C20	0.0406 (17)	0.0381 (16)	0.0467 (17)	0.0015 (13)	0.0209 (14)	0.0029 (13)
C21	0.059 (2)	0.053 (2)	0.056 (2)	−0.0073 (17)	0.0149 (17)	−0.0087 (17)
C22	0.080 (3)	0.074 (3)	0.086 (3)	−0.036 (2)	0.020 (2)	−0.016 (2)
C23	0.098 (3)	0.047 (2)	0.101 (3)	0.008 (2)	0.042 (3)	−0.010 (2)
C24	0.068 (2)	0.0397 (18)	0.078 (2)	−0.0062 (17)	0.028 (2)	−0.0051 (17)
O1W	0.177 (4)	0.085 (2)	0.090 (2)	−0.048 (2)	0.066 (3)	−0.0119 (18)

### Geometric parameters (Å, º)

**Table:**

O1—C4	1.366 (3)	C11—H11A	0.9700
O1—C24	1.433 (4)	C11—H11B	0.9700
O2—C5	1.350 (3)	C12—C13	1.517 (5)
O2—H2'	0.846 (10)	C12—H12A	0.9700
O3—C20	1.341 (3)	C12—H12B	0.9700
O3—H3'	0.850 (10)	C13—H13A	0.9700
O4—C19	1.375 (3)	C13—H13B	0.9700
O4—C21	1.430 (4)	C14—C15	1.442 (4)
N1—C7	1.272 (3)	C14—H14	0.9300
N1—C8	1.465 (4)	C15—C20	1.401 (4)
N2—C14	1.278 (4)	C15—C16	1.402 (4)
N2—C9	1.461 (4)	C16—C17	1.363 (5)
C1—C2	1.373 (4)	C16—H16	0.9300
C1—C6	1.392 (4)	C17—C18	1.389 (5)
C1—H1	0.9300	C17—H17	0.9300
C2—C3	1.388 (4)	C18—C19	1.377 (4)
C2—H2	0.9300	C18—H18	0.9300
C3—C4	1.377 (4)	C19—C20	1.404 (4)
C3—H3	0.9300	C21—C22	1.501 (5)
C4—C5	1.405 (4)	C21—H21A	0.9700
C5—C6	1.394 (4)	C21—H21B	0.9700
C6—C7	1.455 (4)	C22—H22A	0.9600
C7—H7A	0.9300	C22—H22B	0.9600
C8—C13	1.522 (5)	C22—H22C	0.9600
C8—C9	1.533 (4)	C23—C24	1.493 (5)
C8—H8	0.9800	C23—H23A	0.9600
C9—C10	1.514 (4)	C23—H23B	0.9600
C9—H9	0.9800	C23—H23C	0.9600
C10—C11	1.519 (4)	C24—H24A	0.9700
C10—H10A	0.9700	C24—H24B	0.9700
C10—H10B	0.9700	O1W—H1A	0.864 (10)
C11—C12	1.524 (5)	O1W—H1B	0.866 (10)
			
C4—O1—C24	117.3 (2)	C11—C12—H12B	109.5
C5—O2—H2'	111 (3)	H12A—C12—H12B	108.1
C20—O3—H3'	105 (3)	C12—C13—C8	112.6 (3)
C19—O4—C21	117.5 (2)	C12—C13—H13A	109.1
C7—N1—C8	119.0 (2)	C8—C13—H13A	109.1
C14—N2—C9	121.5 (3)	C12—C13—H13B	109.1
C2—C1—C6	120.5 (3)	C8—C13—H13B	109.1
C2—C1—H1	119.8	H13A—C13—H13B	107.8
C6—C1—H1	119.8	N2—C14—C15	121.8 (3)
C1—C2—C3	120.2 (3)	N2—C14—H14	119.1
C1—C2—H2	119.9	C15—C14—H14	119.1
C3—C2—H2	119.9	C20—C15—C16	119.7 (3)
C4—C3—C2	120.4 (3)	C20—C15—C14	119.8 (3)
C4—C3—H3	119.8	C16—C15—C14	120.4 (3)
C2—C3—H3	119.8	C17—C16—C15	120.2 (3)
O1—C4—C3	125.2 (3)	C17—C16—H16	119.9
O1—C4—C5	115.2 (2)	C15—C16—H16	119.9
C3—C4—C5	119.6 (3)	C16—C17—C18	120.5 (3)
O2—C5—C6	122.0 (3)	C16—C17—H17	119.8
O2—C5—C4	118.1 (2)	C18—C17—H17	119.8
C6—C5—C4	119.8 (2)	C19—C18—C17	120.6 (3)
C1—C6—C5	119.4 (3)	C19—C18—H18	119.7
C1—C6—C7	120.1 (3)	C17—C18—H18	119.7
C5—C6—C7	120.4 (2)	O4—C19—C18	125.4 (3)
N1—C7—C6	122.2 (3)	O4—C19—C20	114.8 (2)
N1—C7—H7A	118.9	C18—C19—C20	119.8 (3)
C6—C7—H7A	118.9	O3—C20—C15	122.3 (3)
N1—C8—C13	111.1 (3)	O3—C20—C19	118.6 (3)
N1—C8—C9	108.2 (2)	C15—C20—C19	119.1 (3)
C13—C8—C9	110.5 (3)	O4—C21—C22	107.2 (3)
N1—C8—H8	109.0	O4—C21—H21A	110.3
C13—C8—H8	109.0	C22—C21—H21A	110.3
C9—C8—H8	109.0	O4—C21—H21B	110.3
N2—C9—C10	110.7 (2)	C22—C21—H21B	110.3
N2—C9—C8	109.2 (2)	H21A—C21—H21B	108.5
C10—C9—C8	111.3 (3)	C21—C22—H22A	109.5
N2—C9—H9	108.6	C21—C22—H22B	109.5
C10—C9—H9	108.6	H22A—C22—H22B	109.5
C8—C9—H9	108.6	C21—C22—H22C	109.5
C9—C10—C11	111.6 (3)	H22A—C22—H22C	109.5
C9—C10—H10A	109.3	H22B—C22—H22C	109.5
C11—C10—H10A	109.3	C24—C23—H23A	109.5
C9—C10—H10B	109.3	C24—C23—H23B	109.5
C11—C10—H10B	109.3	H23A—C23—H23B	109.5
H10A—C10—H10B	108.0	C24—C23—H23C	109.5
C10—C11—C12	110.9 (3)	H23A—C23—H23C	109.5
C10—C11—H11A	109.5	H23B—C23—H23C	109.5
C12—C11—H11A	109.5	O1—C24—C23	107.1 (3)
C10—C11—H11B	109.5	O1—C24—H24A	110.3
C12—C11—H11B	109.5	C23—C24—H24A	110.3
H11A—C11—H11B	108.0	O1—C24—H24B	110.3
C13—C12—C11	110.6 (3)	C23—C24—H24B	110.3
C13—C12—H12A	109.5	H24A—C24—H24B	108.6
C11—C12—H12A	109.5	H1A—O1W—H1B	103 (2)
C13—C12—H12B	109.5		
			
C6—C1—C2—C3	−1.1 (5)	C8—C9—C10—C11	−56.0 (3)
C1—C2—C3—C4	1.7 (5)	C9—C10—C11—C12	56.5 (4)
C24—O1—C4—C3	6.5 (4)	C10—C11—C12—C13	−55.5 (4)
C24—O1—C4—C5	−172.9 (3)	C11—C12—C13—C8	55.4 (4)
C2—C3—C4—O1	−179.7 (3)	N1—C8—C13—C12	65.6 (3)
C2—C3—C4—C5	−0.2 (5)	C9—C8—C13—C12	−54.6 (4)
O1—C4—C5—O2	−0.5 (4)	C9—N2—C14—C15	177.9 (3)
C3—C4—C5—O2	−180.0 (3)	N2—C14—C15—C20	1.4 (5)
O1—C4—C5—C6	177.6 (3)	N2—C14—C15—C16	−177.0 (3)
C3—C4—C5—C6	−1.9 (4)	C20—C15—C16—C17	0.9 (5)
C2—C1—C6—C5	−1.0 (5)	C14—C15—C16—C17	179.3 (3)
C2—C1—C6—C7	176.1 (3)	C15—C16—C17—C18	0.9 (6)
O2—C5—C6—C1	−179.5 (3)	C16—C17—C18—C19	−0.3 (5)
C4—C5—C6—C1	2.5 (4)	C21—O4—C19—C18	−3.8 (4)
O2—C5—C6—C7	3.5 (4)	C21—O4—C19—C20	177.3 (3)
C4—C5—C6—C7	−174.6 (3)	C17—C18—C19—O4	179.0 (3)
C8—N1—C7—C6	176.8 (3)	C17—C18—C19—C20	−2.2 (5)
C1—C6—C7—N1	176.8 (3)	C16—C15—C20—O3	178.3 (3)
C5—C6—C7—N1	−6.2 (4)	C14—C15—C20—O3	−0.1 (4)
C7—N1—C8—C13	102.7 (3)	C16—C15—C20—C19	−3.3 (4)
C7—N1—C8—C9	−135.8 (3)	C14—C15—C20—C19	178.2 (3)
C14—N2—C9—C10	−89.5 (3)	O4—C19—C20—O3	1.4 (4)
C14—N2—C9—C8	147.7 (3)	C18—C19—C20—O3	−177.6 (3)
N1—C8—C9—N2	54.9 (3)	O4—C19—C20—C15	−177.1 (2)
C13—C8—C9—N2	176.7 (3)	C18—C19—C20—C15	4.0 (4)
N1—C8—C9—C10	−67.5 (3)	C19—O4—C21—C22	−178.9 (3)
C13—C8—C9—C10	54.3 (3)	C4—O1—C24—C23	176.0 (3)
N2—C9—C10—C11	−177.6 (3)		

### Hydrogen-bond geometry (Å, º)

Cg is the centroid of the C15–C20 ring.

**Table:**

*D*—H···*A*	*D*—H	H···*A*	*D*···*A*	*D*—H···*A*
O3—H3′···N2	0.85 (1)	1.77 (3)	2.550 (4)	152 (3)
O2—H2′···N1	0.84 (1)	1.85 (2)	2.584 (3)	144 (4)
O1*W*—H1*B*···O3	0.86 (5)	2.34 (7)	3.005 (5)	134 (8)
O1*W*—H1*B*···O4	0.86 (5)	2.38 (8)	3.052 (5)	135 (6)
C21—H21*B*···*Cg*	0.97	2.92	3.810 (4)	153
